# Challenges and effectiveness of remote neurological follow-up of children with concussion following TBI using telemedicine

**DOI:** 10.1007/s11845-024-03862-8

**Published:** 2025-01-21

**Authors:** Türker Demirtakan, Semra Işık, Tugay Usta, Ahmed Edizer, Serkan Doğan

**Affiliations:** 1 Emergency Department, University of Health Science, Taksim Research and Training Hospital, Istanbul, Turkey; 2 Neurosurgery, University of Health Science, Ümraniye Research and Training Hospital, Istanbul, Turkey; 3 Emergency Department, University of Health Science, Kanuni Sultan Süleyman Research and Training Hospital, Istanbul, Turkey

**Keywords:** Concussion, PECARN, Pediatric head trauma, Telemedicine, Video-call visits

## Abstract

**Background:**

Traumatic brain injury (TBI) in children, including concussion, is one of the major causes of emergency department (ED) registration and a significant burden on the health system.

**Objectives:**

The primary goal of this study was to evaluate the outcomes of a telemedicine strategy for remotely monitoring the children with traumatic brain concussions, focusing on their neurological symptoms and signs. The secondary goal was to explore socioeconomic and educational differences among the participating families.

**Methods:**

This study was conducted in a prospective and observational fashion. It included children aged between 6 and 18 years who presented in the ED with head trauma and were subsequently diagnosed with a brain concussion. Enrolled patients split into *telemedicine-only* and *telemedicine* + *readmission* groups according to their concussion symptoms during video-call visits.

**Results:**

We recruited 29 children and performed 75 telehealth visits. Four children were called for readmission, and they comprised the *telemedicine* + *readmission* group. The *telemedicine-only* group included 25 children whose follow-ups were completed remotely. The median PECARN score was 1 (IQR = 0.75), and the most common reason for head trauma was simple falls from the same level (*n* = 18, 62%); 22 (76%) children were suffering from headaches; 55% of the families were in very low-income status. During the video-call visit sessions, three children stated worse headaches, and one child’s parents reported consistent sleepiness.

**Conclusion:**

This study demonstrates the potential effectiveness of telemedicine in monitoring children with concussions, especially in regions with diverse socioeconomic backgrounds and overcrowded metropolitan hospitals.

## Introduction

Head trauma in children is a major cause of emergency department (ED) registration and a significant burden on the health system. Traumatic brain injury (TBI) is a broad term for brain damage caused by external forces such as a bump, blow, jolt, or penetrating injury to the head [1]. The global incidence of TBI is estimated to be 47–280 per 100.00 and is increasing [2–4]. Over 80% of pediatric TBI cases are considered mild (mTBI), which is commonly defined by a brief loss of consciousness or a period of confusion and amnesia with negative head computed tomography (CT) findings. The mTBI spectrum also involves concussions. Concussions are a type of TBI caused by biomechanical forces, typically without visible findings on conventional neuroimaging scans. Our definition of concussion aligns with the Consensus Statement on Concussion in Sport from the 5th International Conference held in Berlin in 2016 [5]. Most children with mTBI or concussion do not require neurosurgical intervention and are discharged from EDs after the completion of their urgent care. However, recent studies have also revealed worse neurological outcomes and mTBI-related disabilities in children with concussions in rural areas and underserved communities owing to challenges in triage, transport, treatment, and loss of follow-up. On the other hand, lower socioeconomic status and lower family income increase neurological disability and mortality risks [6, 7].

The healthcare landscape has been rapidly evolving, and the adoption of telehealth technologies offers new approaches to manage emergency cases [8]. Overcrowding in the ED has been associated with several adverse consequences, including treatment delays and patient mortality [9, 10]. The overwhelmed patient surge during the COVID-19 pandemic caused dramatic disruption in the healthcare system. Telemedicine, particularly tele-critical care, is achieved to enhance a hospital’s clinical capabilities through providing remote patient monitoring. The National Emergency Tele-Critical Care Network project in the USA has shown that establishing a national emergency telemedicine system is practical and could provide additional support to the healthcare system in times of need, specifically by increasing bed availability, optimizing hospital admissions, and decreasing patient transfers to congested referral centers [11]. Telemedicine will also play a key role in trauma care and management in the future. Trauma resuscitation can be performed successfully and safely under trauma experts’ remote supervision using telehealth technologies [12].

This study explores the unclear field of remote neurological follow-up, particularly in the Turkish healthcare system, for children diagnosed with concussions following head trauma. In crowded hospitals in overpopulated metropolitan areas in Istanbul, it can be challenging to follow up the patients who have suffered from TBI. In these circumstances, telemedicine can be used for following up these patients. We investigated the innovative use of telehealth approaches to meet the clinical requirements of children presenting with concussions after head trauma. The primary goal of this study was to evaluate the outcomes of a telemedicine strategy for remotely monitoring children with traumatic brain concussions, focusing on their neurological symptoms and signs. The secondary goal was to explore socioeconomic and educational differences among the participating families.

## Methodology

### Study design and settings

This prospective and observational pilot study was conducted in the ED of a tertiary care referral center for the western part of Istanbul. Approximately 500 patients with various types of trauma receive emergency health care in 24 h. In our ED, 40% of the total admissions are under 18 years of age, and 30% of these children have diverse head traumas. Emergency physicians and neurosurgeons manage those children with good coordination and communication skills. Our conventional strategy for children with mTBI is early discharge and parental education about neurological monitoring at home. In this study, we performed remote neurological monitoring of children with concussions using telemedicine via a video-call application. This is the first telehealth experience for children with concussions in our country.

### Ethical approval

The study protocol followed the Helsinki Protocol. We presented informed consent forms to both the children and their legal caregivers. Informed consent for children was obtained by explaining the procedure in an age-appropriate language and seeking the child’s agreement to participate. The study was ethically approved by the institutional ethics committee of our university (approval number: KAEK/2022.02.43).

### Selection of participants

Participants were recruited by emergency medicine physicians and neurosurgeons between July 2022 and February 2023. Each week, clinicians selected one child based on predefined criteria. The inclusion criteria included patients aged 6 to 18 years who presented to the ED with head trauma, had a PECARN score of ≥ 1, and were subsequently diagnosed with a concussion. This age range was determined to provide optimal communication and coordination during the video-call visits.

Patients with severe head trauma, such as skull fractures, subarachnoid hemorrhage, subdural or epidural hematoma, or pneumocephalus, were not included in the study. Those with a PECARN score of 0 and no concussion symptoms were also excluded. Additionally, individuals younger than 6 years or older than 18 years of age, as well as those (or their legal caregivers) who did not provide informed consent, were also excluded.

### Pediatric head trauma management in the ED

Deciding whether to perform a head CT scan is a challenge for clinicians when encountering a child with head trauma. PECARN score was applied to determine the severity and to make a decision to perform a CT scan. The PECARN score was created by Kupperman et al. in 2009 to assist clinicians in identifying children with head injuries requiring CT scans based on their risk of significant intracranial injury. These rules include variations between children aged < 2 years and those aged ≥ 2 years. Clinical signs, based on their presence or absence, aid in determining whether to conduct a head CT scan [13]. In this study, the PECARN rules for > 2-year-old children played a key role in shaping the patient management strategy. We followed this strategy: If a patient received 0 from the PECARN rules, they were hosted for at least 2 h of neurological follow-up in the ED without a CT scan. If there are no new neurological findings or symptoms, we consider discharging patients by educating parents to follow the children’s neurological and mental status at home. If a patient received 1 or more from PECARN rules, we consulted with a neurosurgeon, and a head CT scan was considered for those patients. The neurosurgeon took over the patient’s management if we detected any traumatic pathology in the skull or brain parenchyma, which involved skull fracture, subarachnoid hemorrhage, epidural or subdural hematoma, pneumocephalus, and cerebral contusion. If we did not detect visible pathology in the skull or brain parenchyma, we considered following their neurological status for at least 6 h in the ED in terms of any new onset or worsening neurological symptoms [14, 15].

**Fig. 1:**
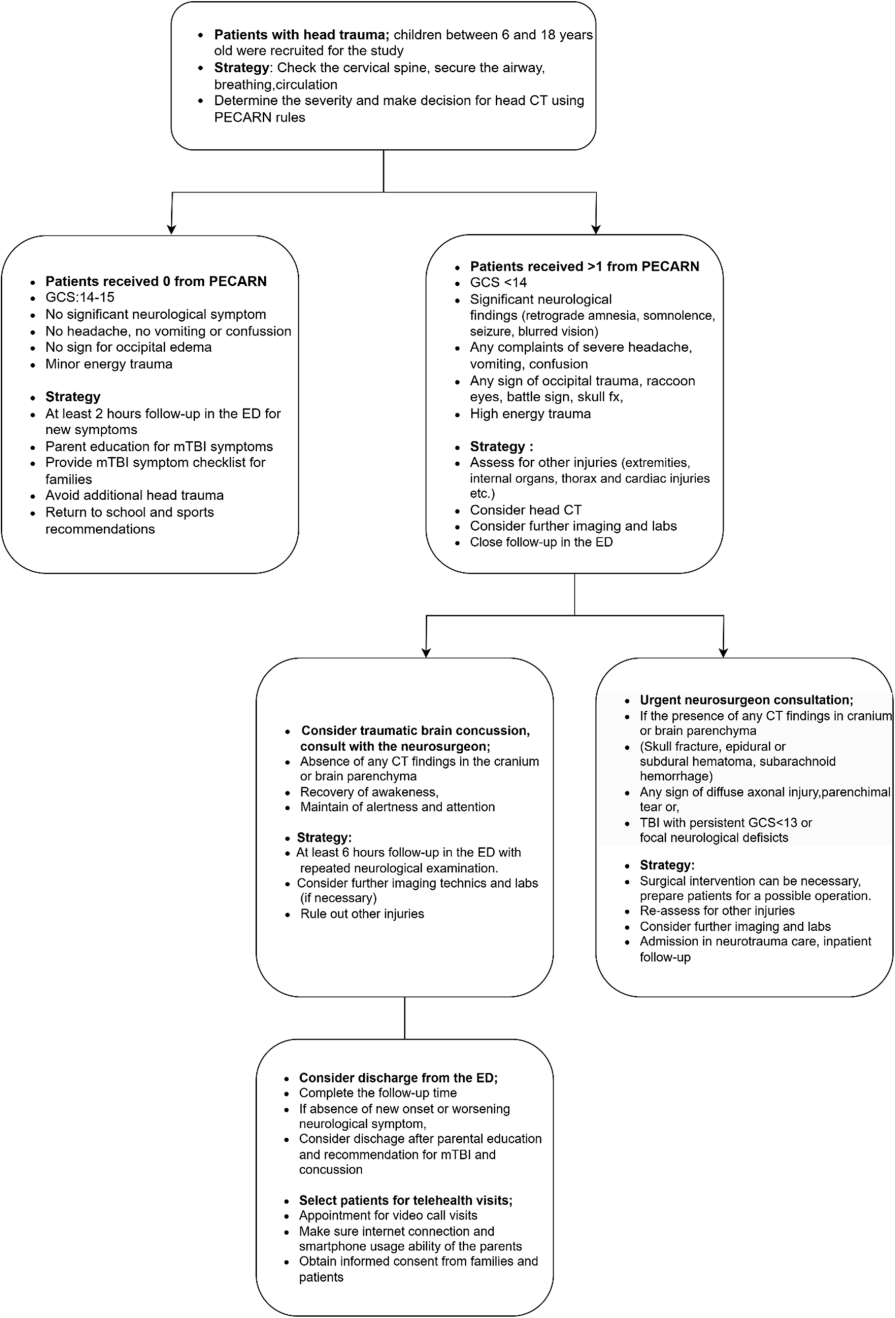
Pediatric head trauma management and patient recruitment strategy

We selected the enrolled patients for remote follow-up and video-call visits among those who received 1 or more points from PECARN rules without remarkable and visible traumatic skull or brain pathology in head CT. The patients were diagnosed with concussion by an ED specialist and a neurosurgeon. We considered releasing these patients from the ED after completing the monitoring time (at least 6 h). Before discharge, the main symptoms and findings of the children were recorded on the patient examination chart. Patients and their parents or legal caregivers were briefly educated about possible TBI symptoms. A brief symptom checklist was provided to the parents to assess the neurological symptoms of their children at home. The checklist included severe headache, repeated vomiting, visual problems, slurred speech, seizures, loss of consciousness, bleeding or fluid leakage from the nose or ears, and black and blue coloration below the eye or behind the ears. We recommended bringing the children to the ED as soon as possible in any unexpected situation or when a new onset sign or symptom on the checklist was observed. We did not recommend going to school for 2 days and returning to play for 1 week to avoid repeated head trauma (Fig. 1) [15, 16].

### Telehealth visits and remote neurological follow-up

The neurological status of the selected patients was checked via video-call sessions. We scheduled three subsequent video-call visits. Our study focused on children aged 6–18 years to ensure optimal cooperation and facilitate a thorough remote neurological examination. Telehealth sessions were conducted using the ZOOM teleconferencing platform [17]. The initial communication was with the parents (or legal caregivers), and the telehealth session with the child proceeded with their consent.

Each telehealth session began with verifying the child’s name, surname, age, school grade, and current date. We then gathered information about the nature of the injury, the trauma mechanism, and their experiences in the hospital. Age-appropriate cognitive tests include counting backward from 10 or 100 in increments of 7 or reciting the months in reverse order. Short-term memory and cognitive functions were assessed using simple and coherent questions. We diligently reassessed the symptoms and complaints presented on the first ED admission. We interrogated their symptoms and complaints with “better,” “worse,” and “no change” questions. Following that, we carried out a neurological assessment that included cranial nerves, motor and sensory exams, gait, and balance, guided by the “Virtual Neurological Exam” by Al Houssana et al. [18].

We also established criteria for readmission to the ED. If a child reported “worse” symptoms or showed failure during any segment of the neurological evaluation, they were promptly recommended for readmission to the ED for an on-site neurological assessment. Persisting concerns and anxiety in parents also served as criteria for readmission in our study.

### Study variables and statistical analysis

Some demographic and clinical specialties were interrogated in participants and families for this study. Age, gender, family structure, education, and monthly incomes of parents, trauma reasons, and PECARN scores were recorded. Patients’ symptoms and clinical findings were recorded on admission and checked during telemedicine visits. On the first admission, recorded signs and symptoms were classified as “better” or “worse” after telemedicine visits, according to the patient’s response. The selected patients were split into two groups. The first group consisted of patients who declared no worsening neurological symptoms, and their follow-up was exclusively conducted through remote video-call visits (*telemedicine-only group*). The second group comprised patients who reported worsening or newly onset neurological symptoms during the video-call visits and subsequently requested readmission to the ED (*telemedicine* + *readmission group*). Appropriate statistical tests were applied to those two groups. Numeric variables were expressed with mean and standard deviation or median value and interquartile range. Mann–Whitney U test was used to compare the numeric variables. Pearson’s chi-square and Fisher’s exact tests were used for independent categorical variables, and the McNemar test was used for dependent categorical variables. All the statistical tests were performed in a 95% confidence interval, and the *p*-value was accepted as 0.05 for the significance.

## Results

We recruited 29 children with concussions (23 male and 6 female) in 8 months. Four children were called for readmission for on-site reassessment, and they comprised the *telemedicine* + *readmission* group. The *telemedicine-only* group included 25 children whose follow-ups were completed remotely via video-call visits. The mean age is 9.65 (StD = 2.66) years, and the minimum and maximum ages are 6 and 15, respectively. The median PECARN score was 1 (IQR = 0.75) in the study; 15 children got the minimum score from PECARN to cover the inclusion criteria; however, one patient got four points because of the severe trauma mechanism. They all underwent head CT scans, and the research team and external radiology experts did not detect any visible traumatic pathology in the brain parenchyma or skull.

The most common cause of head trauma was simple falls at the same level, accounting for 18 cases (62%). Six children (21%) were injured during sports activities. Other causes included car accidents (three cases, 10%) and violence (two cases, 7%). Severe headache was the most common complaint, reported by 22 children (76%), followed by dizziness and drowsiness in 10 children (35%). Occipital scalp edema was the most frequent physical examination finding, present in seven children (24%), while retrograde amnesia was noted in six children (21%).

The socioeconomic and education levels of the families were also assessed; 55% of the families had low-income status (minimum wage or less monthly). Only four families had at least one member with a Bachelor’s degree. In this study, one family member was illiterate, and 21 family members had only literacy or elementary-level education. There were no significant differences in PECARN scores, trauma types, socioeconomic levels, or educational grades between the two groups (Table 1).

**Table 1:** Demographic and clinical characteristics of the children with concussion

Patient characteristics	*Total follow-up* *(n* = *29)*	*Only telemedicine* *(n* = *25)*	*Telemedicine* + *readmission* *(n* = *4)*	*p-value*
Age (years), *Mean* ± *StD, min – max*	9,65 ± 2,66 (6–15)	9,72 ± 2,73 (7–15)	9,25 ± 2,5 (6–12)	> 0.05*
Gender, male	23 (79%)	20 (80%)	3 (75%)	> 0.05†
Gender, female	6 (21%)	5 (20%)	1 (25%)	> 0.05†
Family type				
• Small	23 (79%)	19 (76%)	4 (100%)	> 0.05†
• Large	4 (14%)	4(16%)	0	-
• Divorced parents	2 (7%)	2 (7%)	0	-
Education level of parents				
• Less than elementary school	12 (41%)	9 (36%)	3 (75%)	> 0.05†
• Intermediate and above	13 (45%)	12 (48%)	1 (25%)	> 0.05†
• Bachelor’s degree	4 (14%)	4 (16%)	0	-
Monthly income				
• Minimum wage or less	16 (55%)	14 (56%)	2 (50%)	> 0.05†
• > Minimum wage	13 (45%)	11 (44%)	2 (50%)	> 0.05†
Trauma types				
• Fall from same level	18 (62%)	15 (60%)	3 (75%)	> 0.05†
• Sports injury	6 (21%)	5 (20%)	1 (25%)	> 0.05†
• Car accident	3 (10%)	3 (12%)	0	-
• Violence	2 (7%)	2 (8%)	0	-
PECARN *(median, IQR)*	1 (0.75)	2 (1)	1.5 (1.5)	> 0.05*
PECARN scores				
• 1	15 (51%)	12 (48%)	3 (75%)	
• 2	11 (38%)	11 (44%)	0	
• 3	2 (7%)	1 (4%)	1 (25%)	
• 4	1 (3%)	1 (4%)	0	

^*^Mann–Whitney U test was used to compare two groups

^†^Chi-square test was used to compare two groups

We performed 75 telehealth visits and four on-site visits with enrolled patients and their parents; 21 children attended all three appointed telehealth visits, and four children participated in only two video-call visits; 19 children who declared headaches on their initial ED admission described their symptoms as “better,” whereas, three children stated “worse” headaches (McNemar test *p* < 0.001). Nine children who had dizziness and drowsiness in the ED appeared awake and active during the video-call visits; however, one child’s parents complained about sleepiness (McNemar test *p* < 0.001) (Table 2). Those patients were re-examined on-site. Additionally, three children who reported worse headaches underwent a follow-up CT scan. No significant neurological deficits were found in their examination findings, nor was any visible parenchymal traumatic pathology detected in the head CT scans. They were discharged with additional medical advice and prescribed analgesic or anti-emetic medications.

**Table 2:** Symptoms and neurological findings following children with concussions using telemedicine

Clinical findings and symptoms	*Presented on admission*^*‡*^	*Telemedicine follow-up*		*Change over time*
		*Better*^*§*^	*Worse*^*||*^	*(p-value)*^*¶*^
Severe headache	22/29 (76%)	19/22 (86%)	3/22 (14%)	** < 0.001**
Dizziness	10/29 (35%)	9/10 (90%)	1/10 (10%)	** < 0.001**
Occipital edema	7 /29 (24%)	6 /7 (86%)	1/7 (14%)	** < 0.001**
Retrograde amnesia	6/29 (21%)	6/6 (100%)	0/6 (0%)	-
Nausea	5/29 (17%)	5/5 (100%)	0/6 (0%)	-
Vomiting	4/29 (14%)	3/4 (75%)	1/4 (25%)	-
Slurred speech	4/29 (14%)	4/4 (100%)	0/4 (0%)	-
Visual problems	0/29	-	-	-
Seizures	0/29	-	-	-
LOC > 5 min	0/29	-	-	-
Rhinnorhea/otorrhea	0/29	-	-	-
Racoon eye	0/29	-	-	-
Battle sign	0/29	-	-	-

^*‡*^Number of patients with related clinical signs or symptoms on admission/total number of enrolled patients

^*§*^Number of patients who report better course in their clinical signs or symptoms on admission/number of patients with related clinical signs or symptoms on admission

^*||*^Number of patients who report worsening in their clinical signs or symptoms on admission/number of patients with related clinical signs or symptoms on admission

^¶^McNemar test was used to compare categorical variables

## Discussion

Since the first ED-based telemedicine was initiated in an off-shore island of Taiwan in 1996, the utilization of telemedicine and remote case management technologies has swiftly increased across the world [19]. Especially during the COVID-19 pandemic, healthcare providers urgently needed alternative routes for communication [20]. This motivation also influenced Turkish health policymakers, leading to the first legislative regulations for telemedicine applications being launched in 2021. According to the literature, this is the first study that focused on remote neurological follow-up for children with a concussion in Turkey. A total of 75 telehealth visits were carried out with 29 patients using an open-access and recordable online videoconference platform. Consistent with recent studies, falls from the same level and sports injuries were the leading causes of mTBI [21]. All the patients met the PECARN head CT rules, and therefore, they underwent head CT on the first ED admission to rule out life-threatening skull and brain injury.

Parents of 12 children had elementary-level education, literacy, or less. Only four children’s families had a Bachelor’s degree. Low-income and socioeconomic level families from the region participated in the study enthusiastically and diligently. Thus, we could observe the participation and cooperation of these demographic groups in innovative implementations of emergency medicine. The study took place in a busy and overcrowded ED in western Istanbul, serving around 200,000 patients of all ages with diverse traumas, from minor injuries to life-threatening cases, annually. The number of ED visits now has been exceeding the yearly population for 15 years in Turkey (2022) [22]. Public health specialists reported that ED overcrowding threatens public health by compromising patient safety and diminishing the reliability of the entire emergency care system [23]. TBI is one of the leading reasons for ED admissions among children and young adults [24]. Using telemedicine for children with mTBI can help physicians, reduce ED overcrowding, and improve healthcare quality [25].

Telemedicine is an evolving field in Turkey. The Picture Archiving and Communication System (PACS) is a widely used system across the country, allowing for the digital storage and sharing of medical images. The integration of telemedicine aligns with Turkey’s healthcare strategy to improve patient care and healthcare infrastructure efficiency [26]. Despite the newly launched regulations, there is still no clear algorithm and national guidelines for telehealth utilization in EDs.

Recent pieces of evidence have shown that telemedicine is a safe and cost-effective method to assist in triage and care for well-selected patients with mTBI/concussional symptoms in follow-up care [27]. Results from the open-pilot study of the SMART program, which was conducted for following mTBI using a novel web-based intervention that combined symptom monitoring, activity self-management, and educational module: Using the SMART program, adolescents with mTBI and their parents self-reported symptom burden and recovery progress over four weeks. The study suggested that this type of web-based management program addresses the call for interventions and offers the advantages of being personalized, low-cost, accessible, and scalable [28, 29].

A further study investigated the feasibility of a novel 6-week telehealth exercise regimen for children in Washington state who had persistent post-concussive symptoms. It was developed due to the limited healthcare access during the COVID-19 pandemic. Nineteen children with post-concussive symptoms participated in the Mobile Subthreshold Exercise Program (MSTEP). This program utilized a digital wristband to track exercise goals and performed weekly virtual teleconferences (ZOOM) for six weeks. The study assessed symptoms, fear avoidance, and quality of life. The findings emphasized the efficiency of telehealth-mediated interventions in concussion recovery [30]. The relationship between pediatric concussion outcomes and socioeconomic status is difficult to predict. *Zonfrillo* et al. conducted a prospective study to reveal the long-term effects of education level and monthly income on pediatric post-concussion. Limited parental education and low income were associated with poorer TBI-related quality of life [31]

Telemedicine services for children with mTBI are commonly employed in underserved northern communities in Canada. Twenty patients with concussion or head trauma were assessed through the *Pan Am Clinic Connect Program* in a remote northern community in Manitoba. Throughout the study, a total of 66 telemedicine encounters occurred, consisting of 57 videoconferencing appointments and 9 telephone follow-ups. In conclusion, 18 out of 20 patients (90%) had met the criteria for clinical recovery, one patient was lost to follow-up, one remained in treatment, and one was discharged under the care of a headache neurologist [27]. *Taylor* et al. shared their one-year retrospective experience of telemedicine utilization for pediatric head trauma cases in Salt Lake City, Utah. In this study, an emergency medicine physician from a partner hospital initiated a consultation request using a computer or smartphone then a pediatric surgeon conducted video conferences to obtain medical history from both patients and their parents. In total, eight patients were assessed. Following successful concussion testing, patients were observed and later discharged from the ED [32].

These important limitations should be considered when the findings of this study are interpreted. First, this study recruited a small cohort of pediatric concussion patients who lived in a metropolitan city. Even though Istanbul, an overpopulated metropolitan city, boasts high-standard trauma centers and hospitals, there has been no telemedicine algorithm established for pediatric trauma patients yet. Nevertheless, the research team set a special remote follow-up algorithm with limited staff just before the beginning of this study. Canadian and American clinicians are efficiently benefiting from telehealth applications through the governmental and publicly funded telemedicine staff and digital applications. For example, the *Pan Am Concussion Program* is a provincial government-funded clinical program in Winnipeg, Canada. This program cooperates with the Winnipeg Regional Health Authority, a government-endorsed organization that oversees the operation of publicly funded healthcare facilities [21, 27]. Our next step is conducting a further study with a larger multicentral research team and a specific telemedicine application. On the other hand, further studies are needed to compare the utilization of urban and rural areas of Turkey. Because there are widely ranged sociodemographic and technological differences, video calls can affect attendance at visits and reduce the benefits of implementing telemedicine [33]. There is also an inherent bias risk of observation studies and consecutive sampling methods. Randomized trials to compare children with concussions between inpatients and remote follow-ups provide more reliable and useful information in this manner.

In conclusion, it was the first telemedicine experience to remote neurological follow-up of children with concussions following TBI in our country. Falls on the same level and sports trauma were the leading reasons for pediatric concussions. We were able to reach all children and educate their families, despite more than half of the families having low educational and socioeconomic levels. This study will provide a novel perspective for setting up a dedicated telemedicine application for triaging and following children with TBI. Precise and clear remote monitoring algorithms must be developed for those children.

## Data Availability

All relevant data are included in this manuscript. Telemedicine records used in this study are retained for six months and then permanently deleted, in accordance with institutional and regulatory guidelines. No additional data or records are available for sharing.

## Abbreviations

ED: Emergency department
mTBI: mild Traumatic brain ınjury
CT: Computed tomography
PECARN: Pediatric emergency care applied research network
StD: Standard deviation
IQR: Interquartile range
